# NTM-Based Skill-Aware Knowledge Tracing for Conjunctive Skills

**DOI:** 10.1155/2022/9153697

**Published:** 2022-07-27

**Authors:** Qiang Huang, Wei Su, Yuantao Sun, Tianyuan Huang, Juntai Shi

**Affiliations:** ^1^School of Information Science and Engineering, Lanzhou University, Lanzhou 730000, China; ^2^Ant Group, Hangzhou, China

## Abstract

Knowledge tracing (KT) is the task of modelling students' knowledge state based on their historical interactions on intelligent tutoring systems. Existing KT models ignore the relevance among the multiple knowledge concepts of a question and characteristics of online tutoring systems. This paper proposes a neural Turing machine-based skill-aware knowledge tracing (NSKT) for conjunctive skills, which can capture the relevance among the knowledge concepts of a question to model students' knowledge state more accurately and to discover more latent relevance among knowledge concepts effectively. We analyze the characteristics of the three real-world KT datasets in depth. Experiments on real-world datasets show that NSKT outperforms the state-of-the-art deep KT models on the AUC of prediction. This paper explores details of the prediction process of NSKT in modelling students' knowledge state, as well as the relevance of knowledge concepts and conditional influences between exercises.

## 1. Introduction

With the development of intelligent tutoring systems (ITSs) and the emergence of massive open online courses (MOOCs) [1, 2], knowledge tracing plays an important role in improving the efficiency of personalized learning platforms. Knowledge tracing is the task of modelling students' knowledge state based on their historical interactions to predict students' mastery of knowledge concepts (KCs), where a KC can be an exercise, a skill, or a concept [3, 4].

In order to better model students' knowledge state, various knowledge-tracing models have been proposed. In previous studies, Bayesian knowledge tracing (BKT) is a powerful knowledge-tracing model. BKT models students' knowledge concept state by using a hidden Markov model (HMM) for each KC [5].

As deep learning develops, a lot of deep learning models have been applied in KT. Chris Piech applies the recurrent neural network (RNN) to model the student learning process for the first time and proposes deep knowledge tracing (DKT) [6–9]. The dynamic key-value memory network (DKVMN) uses a static memory called key and a dynamic memory called value to discover latent relations between exercises and knowledge concepts [10, 11]. Self-attentive knowledge tracing (SAKT) proposes a self-attention-based KT model to model the students' knowledge state, with exercises as attention queries and students' past interactions as attention keys/values [3, 12–15].

However, the aforementioned works only focus on students' exercise interactions and ignore the relations between questions and skills. It cannot model students' knowledge state accurately by merely focusing on students' interactions. Knowledge tracing models begin to pay attention to the structure of the knowledge concepts [16–18].

Deep hierarchical knowledge tracing models students' knowledge state by capturing the hierarchical structure of questions and knowledge concepts [16]. Neil Heffernan's latest work considers the question information to which the knowledge concept belongs [17]. Graph-based knowledge tracing considers the influence among neighboring knowledge concepts [19–22]. The bipartite graph is an effective structural model to capture latent relations between questions and skills [18]. This method is effective, but the amount of calculation is huge because it needs to extract questions and skills, respectively. Thus, it is difficult to be regarded as a streamlined and effective knowledge-tracing model.

None of the above KT models make full use of the multiknowledge concept information of the questions. Existing knowledge tracing models cannot capture latent relations between questions and concepts concisely and effectively. We know that questions are generally composed of multiple knowledge concepts, which are actually closely related. In order to better model the students' learning process, our model is constructed by using neural Turing machines (NTMs), which are an instance of memory-augmented neural networks (MANNs) that have a large external memory capacity [23–25]. Therefore, on the basis of above deep knowledge tracing models, we propose an NTM-based skill-aware knowledge-tracing model. The highlight of our work is to utilize the knowledge concept composition information of questions to model the students' knowledge state more accurately and to discover more latent relevance among knowledge concepts effectively. The contributions of this paper are concluded as follows:

- We process the real-world KT datasets in detail and discover new characteristics of online tutoring systems and knowledge tracing datasets.
- We design a question-skill dictionary algorithm to obtain the conjunctive skills of questions. The input encoding contains both students' answering interaction information and the related knowledge concept information.
- We apply neural Turing machines into knowledge tracing innovatively to enhance the memory capacity of our model and to predict students' mastery of knowledge concepts accurately and discover knowledge concept substructure effectively.
- We propose a novel NTM-based skill-aware knowledge-tracing model for conjunctive skills and apply a novel loss optimization function to deep knowledge tracing to enhance the model's ability of skill awareness. Our model considers the conjunctive knowledge concept information contained in a question in the process of modelling the students' knowledge state; thus, our model outperforms existing KT models.

The rest of this paper is organized as follows: Section 2 presents a brief overview of related work in the field of knowledge tracing. In Section 3, we formulate the process for NSKT to perform the knowledge-tracing task. Then, Section 4 introduces the characteristics and classifications of online tutoring systems. The details of the NSKT model are provided in Section 5. The experimental results and the comparison of models' performance in the real-world datasets are given in Section 6. In Section 7, we discuss in detail the process of NSKT in modelling the students' knowledge state.  Section 8 presents the conclusions and future studies of this work.

## 2. Related Work

In this section, we present a brief overview of the models and methods of related work in the field of knowledge tracing, which can be classified into two main categories, as shown in Table 1.

### 2.1. Item Response Theory

Item response theory is the most commonly used cognitive model to predict students' mastery of knowledge concepts before knowledge tracing was proposed in 1995 [26, 27]. On the basis of IRT, the students' knowledge state cognitive model based on factor analysis was later proposed: LFA [28] and PFA [29]. These logistic regression models predict students' mastery of knowledge concepts by analyzing the relationship among factors that have an impact on students' answering accuracy [30, 31].

### 2.2. Knowledge Tracing

Bayesian knowledge tracing (BKT) models the students' knowledge state by using the hidden Markov model (HMM) for a single knowledge concept, which is represented as a set of binary latent variables [5].

With the rise of deep learning, deep knowledge tracing (DKT) was proposed in [6], which regards students' historical interactions as time sequences and models the students' knowledge state by the recurrent neural network (RNN). The experimental results show that DKT has the powerful ability of modelling the students' knowledge state. After DKT, a lot of deep KT models have been proposed to improve the AUC of the prediction of students' mastery of knowledge concepts. However, most of these deep knowledge-tracing models only focus on students' interactions on knowledge concepts and ignore the structural relationship between questions and knowledge concepts.

### 2.3. Question-KC Relation in Knowledge Tracing

Cen et al. proposed the two IRT models (additive factor model (AFM) and conjunctive factor model (CFM)) to model the conjunctive skills in the student datasets [32]. Both the AFM and CFM consider the conjunctive skills information contained in an item to predict the probability of students answering the item correctly.

Deep hierarchical knowledge tracing begins (DHKT) to focus on the hierarchical relationship between knowledge concepts and questions to predict the performance of students [16]. DHKT trains a question embedding by the average embeddings of the skills belonging to the question. The model using the bipartite graphs can capture relationships between knowledge concepts and questions effectively and systematically to pretrain question embeddings for each question [18]. Neil Heffernan's latest work begins to focus on the architecture of knowledge concepts and questions too [17].

## 3. Problem Formulation

Generally, KT can be formulated as a supervised sequence learning problem: the student's interaction tuple at the timestamp *t*, *h*_*t*_=(*q*_*t*_, *a*_*t*_) that represents the combination of which skill (exercise) was answered and if the skill was answered correctly, so *a*_*t*_ ∈ {0,1}, *q*_*t*_ ∈ {*q*_*i*_}_*i*=1_^*M*^, where *M* is the number of unique exercises in datasets. Given the student's past exercise interactions, *H*_*t*_={*h*_0_,…, *h*_*t*_}, the goal of KT is to predict the probability that the student will answer question *q*_*t*+1_ correctly at the next timestamp *t*+1, *P*(*a*_*t*+1_=1/*q*_*t*+1_, *H*_*t*_) [3, 6, 10].

It can be seen that existing KT models only focus on students' exercise interactions, so they are difficult to predict students' mastery of skills effectively. The notations used in this paper are shown in Table 2.

**Definition 1 .**

Related knowledge concepts (RKCs): the related knowledge concepts (RKCs) refer the other knowledge concepts *S* that compose the question *p* with a knowledge concept *q*, where *S* and *q* are mutual conjunctive knowledge concepts (skills).

**Figure 1:**
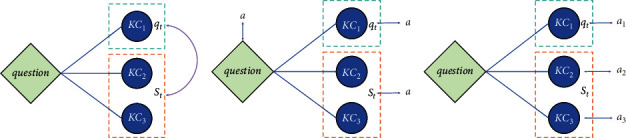
Illustrations. (a) Illustration of RKC *S* related to KC *q*, where *S* and *q* are mutual conjunctive skills. (b) Illustration of the question-level online tutoring system, where a denotes answer correctness to a question. (c) Illustration of the skill-level online tutoring system, *a*_1_ denotes answer correctness to KC1, *a*_2_ denotes answer correctness to KC_2_, and so on *a*_3_.

The Algorithm 1 processes the skills and the questions of the dataset to obtain a dictionary Dic with the question number as the key and conjunctive skills of the question as the value, while conjunctive skills are the skills that make up the same question. The time complexity of Algorithm 1 is *𝒪*(*n*^2^). In this paper, we use KC shown in Table 2 to represent skill. Let *S* be the RKCs related to KC *q* of the answering question *p*, where *S*={*x*/*x* ∈ Dic_*p*_, *x* ≠ *q*}is illustrated in Figure 1(a).

The skill-aware knowledge tracing model can be formulated as follows: the student's interaction at the timestamp *t*, *h*_*t*_′=(*p*_*t*_, *q*_*t*_, *a*_*t*_, *S*_*t*_, *c*_*t*_), where *a*_*t*_ is the correctness to the question *p*_*t*_ on skill *q*_*t*_, *S*_*t*_ are the of RKCs of KC *q*_*t*_, *c*_*t*_ is the correctness to RKCs *S*_*t*_. Given the student's past interactions, *H*_*t*_′=(*h*_0_′,…, *h*_*t*_′), we can predict the probability that the student will answer next KC *q*_*t*+1_ correctly at the timestamp *t*+1, *P*(*q*_*t*+1_)=*P*(*a*_*t*+1_=1/*q*_*t*+1_, *H*_*t*_′) or predict students' mastery of holistic knowledge concepts, {*P*(*q*_*i*_)}_*i*=1_^*M*^.

## 4. Online Tutoring Systems

The online tutoring systems can be classified into two categories:

### 4.1. Question-Level Online Tutoring Systems

In question-level online tutoring systems, students answer the question directly. If the question is answered correctly or incorrectly, all KCs (skills) of the question are answered correctly or incorrectly too. So if a student has answered *q*_*t*_ correctly or incorrectly, then they must answer the RKCs *S*_*t*_ correctly or incorrectly too, which is illustrated in Figure 1(b). Because *q*_*t*_ and *S*_*t*_ are from the same question, so in question-level online tutoring systems, for a student's interaction at the timestamp *t*: (*p*_*t*_, *q*_*t*_, *a*_*t*_, *S*_*t*_, *c*_*t*_),

(1) $$\begin{array}{c} c_{t}=a_{t}. \end{array}$$

### 4.2. Skill-Level Online Tutoring Systems

The question-answering situation in skill-level online tutoring systems is much more complicated than that of the question-level online tutoring system. Students can individually answer one of the skills in the question and can answer this skill once or multiple times. So if a student answers *KC*_1_ correctly, it does not mean that the student must answer *KC*_2_ correctly, which is shown in Figure 1(c).

Superficially, there is no obvious answering correctness relationship between skill *q*_*t*_ and the related skill set *S*_*t*_. However, there are a large number of students answering examples shown in Table 3 in skill-level online tutoring systems, indicating that if a student answers *q*_*t*_ incorrectly many times, even if he finally answers *q*_*t*_ correctly, which demonstrates that his mastery of skill *q*_*t*_ is very poor, and similarly, he has poor mastery of *S*_*t*_. It is very likely that he will answer *q*_*t*_'s-related skills *S*_*t*_ incorrectly. So the student's mastery of *q*_*t*_, *P*(*q*_*t*_) and the student's mastery of *S*_*t*_, *P*(*S*_*t*_) are close:

(2) $$\begin{array}{c} P\left(q_{t}\right)\approx P\left(S_{t}\right). \end{array}$$

This finding is strongly supported by the actual responses of students in skill-level online tutoring systems. So in skill-level online tutoring systems, according to formula (2), we can assume.

(3) $$\begin{array}{c} c_{t}\approx a_{t}, \end{array}$$

as shown in Table 4.

## 5. Method

**Figure 2:**
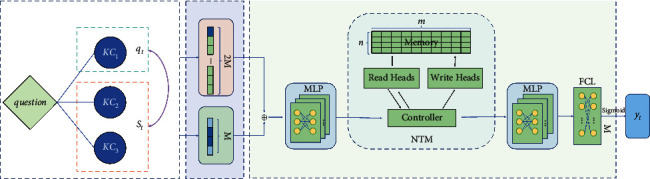
The NSKT framework overview.

In this section, we will give a detailed introduction of our NSKT framework, of which, the overview architecture is given in Figure 2.

### 5.1. Model

The model consists of an encoding layer and a neural network layer. In order to better model the students' knowledge state, the model is constructed with the neural Turing machine, which is an instance of memory-augmented neural networks (MANNs) that offer the ability to quickly encode and retrieve new information [23].

### 5.2. Input Features

#### 5.2.1. Answer Information Encoding

Let *E*^*q*^ be the encoding of the student's interaction tuple (*q*, *a*), thus *E*^*q*^=[*e*_*i*_^*q*^] ∈ {0,1}^2*M*^:

(4) $$\begin{array}{c} e_{i}^{q}=\left\{\begin{array}{cc} 1, & \text{if}i=q+a\times M,\\ 0, & \text{otherwise} \end{array}\right. \end{array}$$

#### 5.2.2. RKC Information Encoding

The information of the set *S* of RKCs related to KC *q* is encoded *E*^*s*^ with a length of *M*: *E*^*s*^=[*e*_*i*_^*s*^] ∈ {0,1}^*M*^:

(5) $$\begin{array}{c} e_{i}^{s}=\left\{\begin{array}{cc} 0 & i\notin S,\\ 1 & i\in S. \end{array}\right. \end{array}$$

### 5.3. Neural Turing Machines

**Figure 3:**
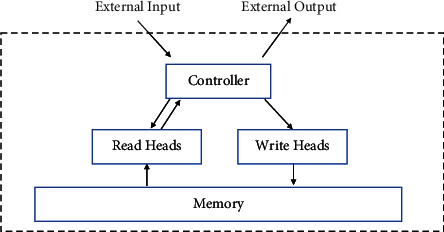
Neural Turing machine architecture.

Neural Turing machines are an instance of memory-augmented neural networks (MANNs) that extend the capabilities of neural networks by coupling them to external memory resources. Experiments show that neural Turing machines have stronger memory capabilities than the LSTM [23], which is very suitable for modeling the students' knowledge state [33–35].  Figure 3 shows a high-level diagram of the neural Turing machine architecture.

As can be seen from Figure 3, the NTM is composed of 4 modules: controller, read heads, write heads, and memory. The controller can be a feed-forward neural network or a recurrent neural network [23, 34] and has read and write heads that access the external memory matrix.

### 5.4. Reading

Let **M**_*t*_ be the external memory content which is a *n* × *m* memory matrix at the timestamp *t*, where *n* is the number of memory locations and *m* is the vector dimension at each memory location. The *n* elements *w*_*t*_(*i*) of **w**_*t*_, which is a vector of weightings over the *n* locations emitted by a read head at the timestamp *t*, obey the following constraints:

(6) $$\begin{array}{c} \sum_{i}w_{t}\left(i\right)=1,0\leq w_{t}\left(i\right)\leq 1,\forall i. \end{array}$$

Let **r**_*t*_ be the read vector of a length *m* returned by the head at the timestamp *t*:

(7) $$\begin{array}{c} r_{t}\leftarrow \sum_{i}w_{t}\left(i\right)M_{t}\left(i\right). \end{array}$$

### 5.5. Writing

The memory matrix **M**_*t*_ at the timestamp *t* is modified by the erase vector **e**_*t*_ and the add vector **a**_*t*_:

(8) $$\begin{array}{c} {\tilde{M}}_{t}\left(i\right)\leftarrow M_{t-1}\left(i\right)\left[1-w_{t}\left(i\right)e_{t}\right]\\ M_{t}\left(i\right)\leftarrow {\tilde{M}}_{t}\left(i\right)+w_{t}\left(i\right)a_{t}. \end{array}$$

### 5.6. Addressing Mechanisms

#### 5.6.1. Focusing on Content

Each head produces a length *m* key vector **k**_*t*_ that is used to compute the normalised weighting *w*_*t*_^*c*^ as follows:

(9) $$\begin{array}{c} w_{t}^{c}\left(i\right)\leftarrow \frac{\exp\left(\left.\beta_{t}K\left[k_{t},M_{t}\left(i\right)\right.\right]\right)}{\sum_{j}\exp\left(\beta_{t}K\left[k_{t},M_{t}\left(j\right)\right]\right)}, \end{array}$$

where *β*_*t*_ is a positive key strength generated by the controller and the similarity measure *K* is cosine similarity:

(10) $$\begin{array}{c} K\left[u,v\right]=\frac{u\cdot v}{\left\|u\right\|\times \left\|v\right\|}. \end{array}$$

#### 5.6.2. Focusing on Location

The location-based addressing mechanism is designed to facilitate both simple iterations across the locations of the memory and random-access jumps. It does so by implementing a rotational shift of a weighting as follows [23].

Firstly, the interpolation gate *g*_*t*_ is used to blend between the weighting **w**_*t*−1_ and the weighting **w**_*t*_^*c*^:

(11) $$\begin{array}{c} w_{t}^{g}\leftarrow g_{t}w_{t}^{c}+\left(1-g_{t}\right)w_{t-1}. \end{array}$$

Furthermore, the model uses a one-dimensional convolution shift kernel to convolve the current weighting *w*_*t*_^*g*^:

(12) $$\begin{array}{c} {\tilde{w}}_{t}\left(i\right)\leftarrow \sum_{j=0}^{n-1}w_{t}^{g}\left(j\right)s_{t}\left(i-j\right), \end{array}$$

where **s**_*t*_ is the shift weighting generated by the controller.

To correct the blur that occurs due to the convolution operation, each head emits one further scalar *γ*_*t*_ ≥ 1 whose effect is to sharpen the final weighting as follows:

(13) $$\begin{array}{c} w_{t}\left(i\right)\leftarrow \frac{{\tilde{w}}_{t}{\left(i\right)}^{\gamma_{t}}}{\sum_{j}{\tilde{w}}_{t}{\left(j\right)}^{\gamma_{t}}}. \end{array}$$

### 5.7. Controller

The NTM controller in our model is the long short-term memory network [36], which can be formulated by the formulas as follows:

(14) $$\begin{array}{c} f_{t}=\sigma \left(w_{f}\left[h_{t-1},x_{t}\right]+b_{f}\right),\\ i_{t}=\sigma \left(w_{i}\left[h_{t-1},x_{t}\right]+b_{i}\right),\\ o_{t}=\sigma \left(w_{o}\left[h_{t-1},x_{t}\right]+b_{o}\right),\\ c_{t}=f_{t}⊙c_{t-1}+i_{t}⊙\;\tanh\left(w_{c}\left[h_{t-1},x_{t}\right]+b_{c}\right),\\ h_{t}=o_{t}⊙\;\tanh\left(c_{t}\right). \end{array}$$

**i**, **f**, **o**, **c**, **h** are the activation matrices of the input gate, the forget gate, the output gate, the memory cell, and the hidden state matrix, respectively. **w** and **b** are the weight matrix and the bias vector of the corresponding gate, respectively. ⊙ denotes the Hadamard product. *σ* and tanh denote the sigmoid and hyperbolic tangent function, respectively:

(15) $$\begin{array}{c} \sigma \left(x\right)=\frac{1}{1+\exp\left(-x\right)}, \end{array}$$

let logits ∈ *ℝ*^*M*^ be the output of the last neural network of the NSKT model, the student's mastery of knowledge concepts predicted by the model at the timestamp *t* is

(16) $$\begin{array}{c} y_{t}=\sigma \left(\text{logits}\right), \end{array}$$

where **y**_*t*_ ∈ *ℝ*^*M*^.

### 5.8. Optimization

The loss function of the model consists of two parts, namely, the answering interaction loss ℒ_1_ and the related knowledge concept information loss ℒ_2_. Let *ℓ* be the binary cross entropy loss:

(17) $$\begin{array}{c} \ell \left(p,a\right)=-\left(a\;\log\;p+\left(1-a\right)\log\left(1-p\right)\right). \end{array}$$

We optimize the average cross entropy loss of the student's interactions as follows:

(18) $$\begin{array}{c} {ℒ}_{1}=\frac{\sum_{t}\ell \left(y_{t}^{T}\delta \left(q_{t+1}\right),a_{t+1}\right)}{\left|H\right|-1}, \end{array}$$

where *δ*(*q*_*t*+1_) is the one-hot encoding of KC *q*_*t*+1_ at the timestamp *t*+1, |*H*| is the total number of the student's interactions, and **T** denotes transpose operation.

The average cross-entropy loss of the related knowledge concept information is

(19) $$\begin{array}{c} {ℒ}_{2}=\frac{\sum_{t}\sum_{i=1}^{\left|S_{t+1}\right|}\ell \left(y_{t}^{T}\delta \left(q_{i}\right),c_{i}\right)}{\sum_{t}\left|S_{t+1}\right|}, \end{array}$$

where *q*_*i*_ ∈ *S*_*t*+1_, *c*_*i*_ is the correctness to skill *q*_*i*_.

The loss for a single student is represented by ℒ, which is as follows:

(20) $$\begin{array}{c} ℒ=\lambda {ℒ}_{1}+\left(1-\lambda \right){ℒ}_{2}\\ =\lambda \frac{\sum_{t}\ell \left(y_{t}^{T}\delta \left(q_{t+1}\right),a_{t+1}\right)}{\left|H\right|-1}+\left(1-\lambda \right)\frac{\sum_{t}\sum_{i=1}^{\left|S_{t+1}\right|}\ell \left(y_{t}^{T}\delta \left(q_{i}\right),c_{i}\right)}{\sum_{t}\left|S_{t+1}\right|}\\ =\sum_{t}\left(\lambda \frac{\ell \left(y_{t}^{T}\delta \left(q_{t+1}\right),a_{t+1}\right)}{\left|H\right|-1}+\left(1-\lambda \right)\frac{\sum_{i=1}^{\left|S_{t+1}\right|}\ell \left(y_{t}^{T}\delta \left(q_{i}\right),c_{i}\right)}{\sum_{t}\left|S_{t+1}\right|}\right), \end{array}$$

where the hyperparameter *λ* is the coefficient that determines the proportion of the answering information loss and the related information loss. We use an optimizer to optimize our model. Let Θ be the minimum of ℒ, thus, the training objective of NSKT is as follows:

(21) $$\begin{array}{c} \Theta \leftarrow \text{optimizer}\left(ℒ\right). \end{array}$$

### 5.9. Skill Awareness

The student's past interactions in online tutoring systems: *H*_*t*_′={*h*_0_′,…, *h*_*t*_′}, where *h*′=(*q*_*t*_, *a*_*t*_, *S*_*t*_, *c*_*t*_) denotes that the student interaction tuple at the timestamp *t*. The set of knowledge concepts Set_*q*_ that students have answered actually so far is represented as follows:

(22) $$\begin{array}{c} Set_{t}^{q}={\left\{q_{i}\right\}}_{i=1}^{t}. \end{array}$$

The set of knowledge concepts (skills) Set^*S*^ answered by NSKT so far is represented as follows:

(23) $$\begin{array}{c} Set_{t}^{S}=S_{1}\cup \ldots \cup S_{t}. \end{array}$$

**Figure 4:**
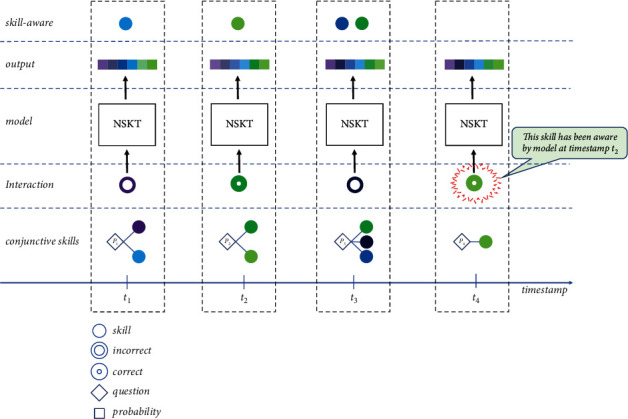
The process of skill awareness in NSKT. Different skills are indicated by different colors.

As shown in Figure 4, when the student answers the next skill *q*_*t*+1_ at the next timestamp *t*+1, even if the student has not answered questions related to skill *q*_*t*+1_ before, *q*_*t*+1_ ∉ Set_*t*_^*q*^, but if NSKT has awareness of skill *q*_*t*+1_ so far, *q*_*t*+1_ ∈ Set_*t*_^*S*^, NSKT can predict the student's mastery of skill *q*_*t*+1_ accurately.

## 6. Experiments

In this section, we give a detailed explanation of datasets and experiments conducted to evaluate the performance of the NSKT model and other KT models in three real-world open-source knowledge tracing datasets.

### 6.1. Datasets

To evaluate KT models' performance, we use three datasets collected from online learning platforms. These three datasets are widely used real-world datasets in KT.

- ASSISTments2009 (https://sites.google.com/site/assistmentsdata/home/2009-2010-assistment-data) (ASSIST09) is provided by the ASSISTment online tutoring platform and is the most widely used dataset in knowledge tracing.
- ASSISTments2017 (https://sites.google.com/view/assistmentsdatamining/dataset/) (ASSIST17) is provided by the 2017 ASSISTments data mining competition and is the latest ASSISTments dataset with the most student responses.
- EdNet (https://github.com/riiid/ednet) is the dataset of all student-system interactions collected over 2 years by Santa, a multiplatform AI tutoring service with more than 780 K users in Korea available through Android, iOS, and Web [37]. We conducted our experiments on EdNet-KT1 which consists of students' question-solving logs and is the record of Santa collected since April 2017 by following the question-response sequence format.

The complete statistical information for the three datasets is shown in Table 5.

The details about the columns in datasets are shown as follows:

ASSISTments:
  - user_id: the ID of the student
  - problem_id: the ID of the problem
  - skill_id: the ID of the skill associated with the problem
  - 1: Correct on the first attempt 0: Incorrect on the first attempt,
EdNet:
  - user_id: the ID of the student.
  - question_id: the ID of the question.
  - tags: the expert-annotated tags for each question.
  - correct_answer: the correct answer of each question recorded as a character between a and *d* inclusively.
  - user_answer: the answer that the student submitted was recorded as a character between a and *d* inclusively.

### 6.2. Dataset Characteristics

- ASSIST09 and EdNet: For multiple skill questions, the records of students' interactions will be repeated with different skill taggings and each record represents the student response to a skill of the question [38].
- ASSIST17: similar to the ASSIST09 dataset, each record in ASSIST17 represents the student response to a skill of the question. However, we noticed the special features of this dataset. A large number of users in the ASSIST17 dataset only answered one skill of multiple skill questions and answered this skill one or more times. The number of multiple skill questions in this situation accounted for 44.88% of the total number of questions answered by students. That is, the student answers one or more skills of multiple skill questions, and the number of responses to a skill may be given once or multiple times.

### 6.3. Compared Models and Implementation Details

To show the performance of our model and demonstrate the improvement of our model to existing KT models, we compare NSKT against the state-of-the-art KT models. We give the reference GitHub repositories of some KT models.

- BKT [5]: Bayesian knowledge tracing uses the hidden Markov model (HMM) to model the students' latent knowledge state as a set of binary variables. We use pyBKT (https://github.com/CAHLR/pyBKT) to implement BKT and set the model parameters: seed=42, num_fits=1.
- DKT-LSTM [6]: the DKT-LSTM is the standard deep knowledge-tracing mode. We implemented DKT (https://github.com/chrispiech/DeepKnowledgeTracing) with the LSTM with tanh activation.
- DKT-NTM: DKT is implemented by using the neural Turing machine (https://github.com/MarkPKCollier/NeuralTuringMachine).
- DKVMN [10]: the DKVMN (https://github.com/jennyzhang0215/DKVMN) is a variation of MANNs, which uses a static memory called key and a dynamic memory called value to model the students' knowledge state.
- SAKT [3]: SAKT (https://github.com/shalini1194/SAKT) is the KT model based on the self-attention architecture with exercises as attention queries and students' past interactions as attention keys/values.
- DSKT: the skill-aware deep knowledge-tracing model is implemented with the LSTM and tanh activation. We dynamically set the value of the coefficient *λ* to explore the best performance of DSKT.
- NSKT: NSKT is an NTM-based skill-aware knowledge tracing. We test the performance of NSKT with different values of the coefficient *λs* to optimize the model's performance.

For all models, we use the Adam optimizer with learning_rate=0.001, beta1=0.9, beta2=0.999, and epsilon=1*e* − 8 to optimize. The minibatch size and the maximum length of the sequence for all datasets are set to 32 and 100, respectively. We perform standard five-fold cross-validation to evaluate all the KT models in this paper. We conduct experiments on the server with an 8-core 2.50 GHz Intel(R) Xeon(R) Platinum 8163 CPU and 64 GB memory.

### 6.4. Experimental Results

#### 6.4.1. Models' Performance

We use the area under the receiver operating characteristic curve (AUC) as an evaluation metric to compare prediction performance among the KT models mentioned in Section 6.3. A higher AUC indicates better performance. The test AUC results in the three real-world datasets for all KT models are shown in Table 6. From the experiment results, we can find the following observations:

- NSKT performs better than the other competing KT models in all datasets and achieves the average test AUC of 85.38%, 82.35%, and 80.81% in ASSIST09, ASSIST17, and EdNet, respectively.
- DSKT performs better than the DKT-LSTM, achieves the average test AUC of 84.88%, 81.27%, and 79.71% in datasets ASSIST09, ASSIST17, and EdNet, respectively, gaining an average performance improvement of 0.82% (DKT-LSTM achieves the AUC of 84.45%, 80.04%, and 78.91%). NSKT performs better than the DKT-NTM, gaining an average performance improvement of 1.33% (DKT-NTM achieves the AUC of 84.53%, 80.51%, and 79.49%).
- The DKT-NTM model has a better performance than the standard DKT-LSTM in knowledge tracing. The DKT-NTM achieves the average test AUC of 84.53%, 80.51%, and 79.49% in the three datasets, respectively, while the standard DKT-LSTM achieves the average test AUC of 84.45%, 80.04%, and 78.91% in the three datasets, respectively.
- The performance of NSKT is better in dataset ASSIST17, which has more complex data features than those of ASSIST09 and EdNet. NSKT gains an average performance improvement of 2.31% in ASSIST17 compared to the standard DKT-LSTM while improving AUC by 0.93% and 0.90% in ASSIST09 and EdNet, respectively. It proves that NSKT is better in mining hidden information from complex educational data features to improve the accuracy of prediction.

**Figure 5:**
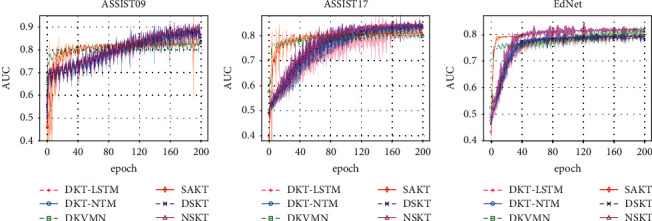
The dynamics for training models in the KT datasets with the best hyperparameter selection for each model and each dataset individually. Each line plots the mean over multiple runs, while the corresponding root mean square error (RMSE) is shown as the shaded area around the mean. (a) ASSIST09. (b) ASSIST17. (c) EdNet.

Figure 5 shows the training process of KT models in the three KT datasets. It shows that the DKVMN and SAKT can learn faster than other KT models. The training speed of the DKT-LSTM, DKT-NTM, DSKT, and NSKT is close, but the test AUC of NSKT is the best.

We set the probability to KC *q*_*t*_ predicted by KT models: *P*(*q*_*t*_), and assume that students will answer KC *q*_*t*_ correctly if *P*(*q*_*t*_) > =0.5 and if *P*(*q*_*t*_) < 0.5, the student will answer *q*_*t*_ incorrectly:

(24) $$\begin{array}{c} a_{t}^{\prime }=\left\{\begin{array}{cc} 0 & P\left(q_{t}\right)<0.5,\\ 1 & P\left(q_{t}\right)>=0.5. \end{array}\right. \end{array}$$

**Figure 6:**
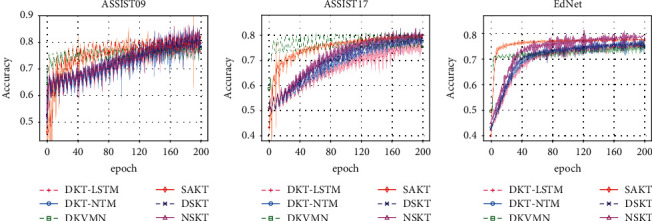
The accuracy of prediction for training models in the KT datasets with the best hyperparameter selection for each model and each dataset individually. Each line plots the mean over multiple runs, while the corresponding root mean square error (RMSE) is shown as the shaded area around the mean. (a) ASSIST09. (b) ASSIST17. (c) EdNet.

If *a*_*t*_′=*a*_*t*_, it means that models can predict correctly. Thus, the accuracy of prediction for KT models in the datasets is shown in Figure 6.

**Figure 7:**
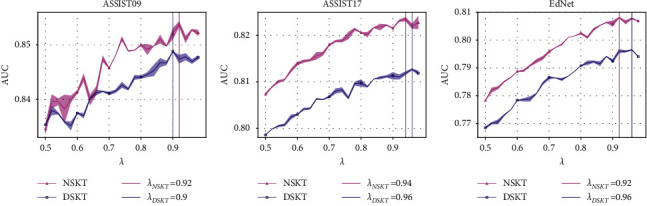
The average test AUC of NSKT and DSKT with different *λ* values in datasets. Each line plots the mean over multiple runs, while the corresponding root mean square error (RMSE) is shown as the shaded area around the mean. (a) ASSIST09. (b) ASSIST17. (c) EdNet.

Figure 7 shows the performance of DSKT and NSKT under different *λ* values and the value of *λ* when models achieve the best performance. From Figure 7, we can draw the following conclusions: the test AUC of DSKT and NSKT is not ideal with a small *λ* value. However, as the value of *λ* increases, the test results of DSKT and NSKT get better and better; thus, we recommend *λ* ≥ 0.9.

### 6.5. Friedman-Aligned Rank Test

We perform the Friedman-aligned rank test [39] on the AUC test results of the KT models shown in Table 6 by the following formula:

(25) $$\begin{array}{c} X^{2}=\frac{12}{nk\left(k+1\right)}\sum R_{i}^{2}-3n\left(k+1\right), \end{array}$$

where *R*_*i*_ is the sum of the ranks of the *i*-th sample, *k* is the number of groups of samples, and *n* is the number of samples in each group. The probability distribution of *X*^2^ can be approximated by that of the chi-squared distribution with *k* − 1 degrees of freedom *χ*_*k*−1_^2^. Now, we test the null hypothesis, which is as follows:

  H_0_: there is no significant difference in the performance of the KT models.

The *P* value *P* of the Friedman-aligned rank test on test AUC results is

(26) $$\begin{array}{c} P\left(\chi_{k-1}^{2}\geq X^{2}\right)=0.013<0.05. \end{array}$$

Then, we reject the null hypothesis H_0_, which indicates a significant difference in the performance of the KT models.

### 6.6. Execution Time

**Figure 8:**
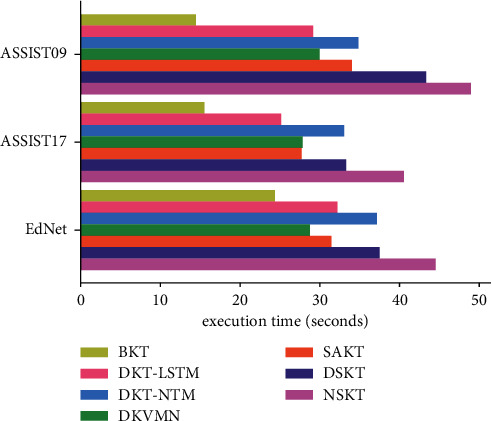
The comparison of the KT models' execution time per 200 batches in each dataset.

We compared the execution time of KT models per 200 batches in each dataset shown in Figure 8. As shown in Figure 8, the BKT model requires the least execution time to train the same size of data. This is because the BKT is not a deep learning knowledge tracing model, and it needs to train fewer parameters. For deep learning knowledge tracing models, the execution times of the DKT-LSTM, DKVMN, and SAKT are close and the execution times of the DKT-NTM and DSKT are close. The execution time of the DKT-NTM is more than that of the DKT-LSTM. The reason can be that the NTM takes more time to access its own external memory matrix. NSKT considers the conjunctive skills of the questions during the training process and needs to access the NTM's external memory matrix to enhance the memory ability of the model. Hence, NSKT has the most execution time, but this is also the reason why NSKT performs better in modelling the students' knowledge state.

The experimental results show that the NTM-based skill-aware knowledge-tracing model has a strong ability to capture the relevance among knowledge concepts and can enhance the model's ability of skill awareness for conjunctive skills and improve the accuracy of prediction in modelling the students' knowledge state. Experiments demonstrate that NSKT is effective.

## 7. Discussion

In this section, we discuss the details of the prediction process of the KT model in modelling the students' knowledge state, as well as the relevance of knowledge concepts and conditional influence between exercises.

### 7.1. Prediction Process

In our opinion, an excellent KT model not only can predict the probability that students will answer questions correctly at the next timestamp accurately but also can perform well in modelling the students' holistic knowledge concept state.

**Figure 9:**
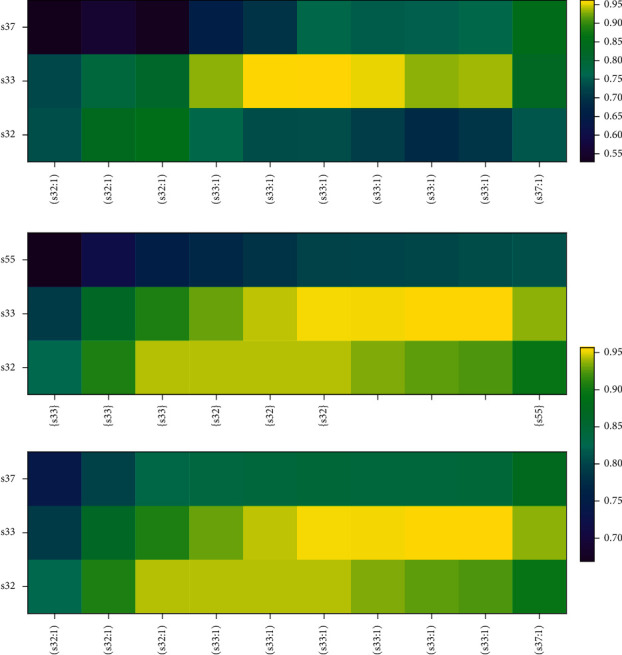
Comparison of the prediction process of *U*_1_ on DKT and NSKT. The color of the heatmap indicates the predicted probability that *U*_1_'s mastery of skills after interaction (*q*_*t*_, *a*_*t*_) at the timestamp *t*. The yellower the color, the higher the probability. (a) Heatmap for the prediction process of DKT. The *x*-axis is the sequence of *U*_1_'s interactions (*q*_*t*_; *a*_*t*_) and the *y*-axis is the skill index. (b) Heatmap for the prediction process of NSKT. The *x*-axis of the lower subfigure is the sequence of *U*_1_'s interactions (*q*_*t*_; *a*_*t*_) and the *y*-axis is the skill index. The *x*-axis of the upper subfigure is the RKC *S*_*t*_ and the *y*-axis is the skill index of the RKCs *S*_*t*_.

Analyzing the prediction process of KT models can show the performance of NSKT. We randomly select a student sample *U*_1_ from the ASSIST09 dataset, and the detailed process of DKT and NSKT modelling *U*_1_'s knowledge state is shown in Figure 9.

It can be seen from Figure 9(a) that although DKT performs fairly well in prediction, DKT only focuses on the knowledge concepts to be predicted at the next timestamp and does not care about the *U*_1_'s mastery of other knowledge concepts. Therefore, after *U*_1_ answers *s*32 correctly (*s*32,1) at the timestamp *t*_3_, the model's predicted probability of *s*32 decreases rapidly, indicating that *U*_1_'s mastery of *s*32 is getting worse and worse, which should not be consistent with the *U*_1_'s real knowledge state shown in Table 7. Because of lacking related knowledge concept (RKC) information, DKT's prediction accuracy and prediction breadth are not ideal.

As shown in Figure 9(b), we use two heatmap subfigures to show the process of modelling the *U*_1_'s knowledge state on NSKT. The *x*-axis of the lower subfigure is the sequence of *U*_1_'s interactions (*q*_*t*_, *a*_*t*_) and the *y*-axis is the skill index. The *x*-axis of the upper subfigure is the RKC *S*_*t*_ and the *y*-axis is the index of the RKCs *S*_*t*_.

Because *U*_1_ answers skill 32 (abbreviated as *s*32) correctly (*s*33,1) in the first three timestamps *t*_1_ − *t*_3_, the predicted probability of *s*32 gets higher and higher and the color of *s*32 in the *y*-axis of the lower subfigure gets brighter and brighter. As shown in the *x*-axis of the upper subfigure, *s*33 is the related knowledge concept of *s*32 in the first three timestamps *t*_1_ − *t*_3_; thus, the predicted probability of *s*33 gets higher and higher and the color of the *s*33 in the *y*-axis of the upper subfigure gets brighter and brighter too.

In the next three timestamps *t*_4_ − *t*_6_, *U*_1_ answers s33 correctly (*s*33,1) in succession, the predicted probability of *s*33 gets higher and higher and the color of *s*33 in the *y*-axis of the lower subfigure gets brighter and brighter. *s*32 is the related knowledge concept of *s*33, so the predicted probability of *s*32 continues to increase, and the color of *s*32 in the *y*-axis of the upper subfigure gets brighter and brighter too and remains at a relatively high value.

In the next three timestamps *t*_7_ − *t*_9_, *U*_1_ continues to answer *s*33 correctly (*s*33,1); however, this *s*33 is a single skill without related knowledge concepts, so only the predicted probability of *s*33 gets higher and higher and the color of *s*33 in the *y*-axis of the lower subfigure gets brighter and brighter.

At the last timestamp *t*_10_, *U*_1_ answer *s*37 correctly (*s*37,1), so the predicted probability of *s*37 gets higher and higher and the color of *s*32 in the *y*-axis of the lower subfigure gets brighter and brighter. Because *s*55 is the related knowledge concept of *s*37, so the predicted probability of *s*55 gets higher and higher and the color of *s*55 in the *y*-axis of the upper subfigure gets brighter and brighter too.

In contrast, we randomly select a student sample *U*_2_ with a low answering accuracy shown in Table 8.

**Figure 10:**
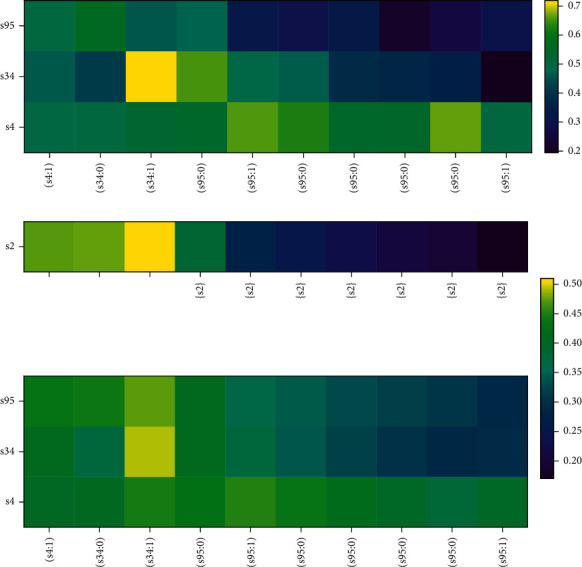
Comparison of the prediction process of *U*_2_ on DKT and NSKT. The color of the heatmap indicates the predicted probability that *U*_2_'s mastery of skills after interaction (*q*_*t*_, *a*_*t*_) at the timestamp *t*. The yellower the color, the higher the probability. (a) Heatmap for the prediction process of DKT. The *x*-axis is the sequence of *U*_2_'s interactions (*q*_*t*_; *a*_*t*_) and the *y*-axis is the skill index. (b) Heatmap for the prediction process of NSKT. The *x*-axis of the lower subfigure is the sequence of *U*_2_'s interactions (*q*_*t*_; *a*_*t*_) and the *y*-axis is the skill index. The *x*-axis of the upper subfigure is the RKC *S*_*t*_ and the *y*-axis is the skill index of the RKCs *S*_*t*_.

The process of DKT and NSKT modelling the *U*_2_'s knowledge state is shown in Figure 10. It can be seen from Figure 10(a) that DKT models the *U*_2_'s knowledge state almost accurately, but the prediction breadth is not enough.

As shown in Figure 10(b), NSKT, like DKT, models the *U*_2_'s knowledge state accurately and performs better in prediction breadth. At the timestamp *t*_4_, *U*_2_ answers *s*95 incorrectly many times, the predicted probability of *s*95 gets lower and lower and the color of *s*95 in the *y*-axis of the lower subfigure gets darker and darker. As shown in the *x*-axis of the upper subfigure, *s*2 is the related knowledge concept of *s*95; thus, the predicted probability of *s*2 gets lower and lower and the color of *s*33 in the *y*-axis of the upper subfigure gets darker and darker too.

It can be concluded from Figures 9 and 10 that NSKT performs better in prediction accuracy and prediction breadth and can better model the students' knowledge state. NSKT not only focuses on students' mastery of the knowledge concept to be predicted at the next timestamp but also focuses on the students' mastery of the related knowledge concepts. This is where NSKT is superior to other existing KT models, and NSKT performs better in modelling the students' knowledge state than DKT [4].

### 7.2. Pearson Correlation Coefficient

In this paper, we use the Pearson correlation coefficient as the metric to measure the correlation among skills. By estimating the covariance and standard deviation of the sample, we can get the sample Pearson coefficient *r*:

(27) $$\begin{array}{c} r_{\left(X,Y\right)}=\frac{\sum_{i=1}^{n}\left(X_{i}-\bar{X}\right)\left(Y_{i}-\bar{Y}\right)}{\sqrt{\sum_{i=1}^{n}{\left(X_{i}-\bar{X}\right)}^{2}}\sqrt{\sum_{i=1}^{n}{\left(Y_{i}-\bar{Y}\right)}^{2}}}. \end{array}$$

**Figure 11:**
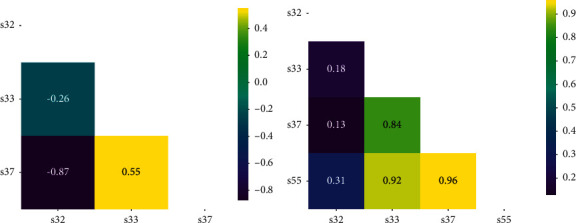
Comparison of *U*_1_'s interaction skill Pearson correlations on DKT and NSKT ((a) DKT and (b) NSKT). Both the *x*-axis and the *y*-axis are the skills in *U*_1_'s interactions.

**Figure 12:**
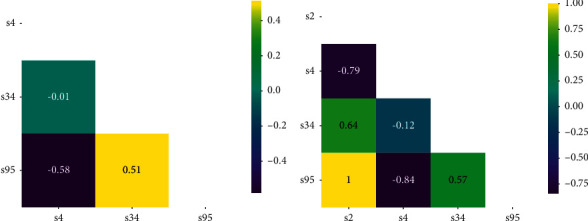
Comparison of *U*_2_'s interaction skill Pearson correlations on DKT and NSKT ((a) DKT and (b) NSKT). Both the *x*-axis and the *y*-axis are the skills in *U*_2_'s interactions.

Figures 11 and 12 show the comparison of skill Pearson correlations of *U*_1_'s interactions and *U*_2_'s interactions on DKT and NSKT, respectively. Figures 11(a) and 12(a) show the skill Pearson correlation on DKT, and Figures 11(b) and 12(b) show the skill Pearson correlation on NSKT. It can be seen from the figures that DKT can only mine the correlation among the skills that have been answered in the past, indicating that DKT cannot effectively discover the relevance among knowledge concepts. As shown in Figures 11(b) and 12(b), NSKT can discover the correlation among four skills, while DKT can only discover among three. For example, it can be seen from Figure 11(b) that the Pearson correlation between *s*32 and *s*55 on NSKT of *U*_1_'s interactions is *r*_(*s*32, *s*55)_ = 0.31, which means there is a weak positive correlation between *s*32 and *s*55.

The Pearson correlation between *s*33 and *s*55 on NSKT of *U*_1_'s interactions is *r*_(*s*33, *s*55)_ = 0.92, which means there is a strong positive correlation between *s*33 and *s*55. Through the above examples, we can conclude that NSKT performs better in the ability of discovering latent relevance among knowledge concepts than existing KT models.

### 7.3. Knowledge Concepts' Discovery

NSKT can learn latent knowledge concept substructure among skills without expert annotations and can cluster related skills into a cluster, which denotes a knowledge concept (KC) class [6].

**Figure 13:**
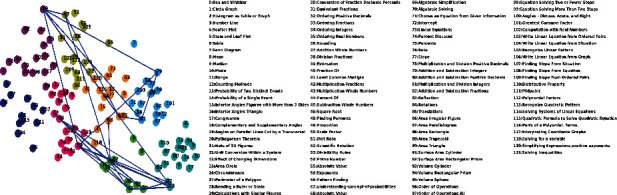
Knowledge concept visualization in ASSIST09 and conditional influences between exercises.

Figure 13 shows the visualization of using k-means to cluster the skill representation vectors, which have been performed by the t-SNE method [40, 41]. All skills are clustered into eight clusters, and each cluster can represent a knowledge concept class. Skills in the same cluster are labeled with the same color, and those skills have strong relevance and similarity. For example, *s*32 and *s*33 do have a strong relevance and similarity because they are very close in Figure 13, which further proves that NSKT has a stronger ability of discovering skill latent relevance information than existing KT models.

We have explored latent conditional influence between exercises by

(28) $$\begin{array}{c} J_{i⟶j}=\frac{y\left(j/i\right)}{\sum_{k}y\left(j/k\right)}, \end{array}$$

where *y*(*j*/*i*) is the correctness probability assigned by NSKT to exercise *j* when exercise *i* is answered correctly in the first time step [6]. We have shown a latent conditional influence relationship among the exercises corresponding to Figure 9(b) interactions. We have marked them with arrow symbols in Figure 13. The line width indicates connection strength, and nodes may be connected in both directions. We only show edges with an influence threshold greater than 0.08. Attached ASSIST09 skill maps are shown in Figure 13 (we only show 110 skills with the skill name).

## 8. Conclusion

In this work, we proposed a novel NTM-based skill-aware knowledge-tracing model for conjunctive skills, which can capture the relevance among the multiple knowledge concepts of questions to predict students' mastery of knowledge concepts (KCs) more accurately and to discover more latent relevance among knowledge concepts effectively. In order to better model the students' knowledge state, we adopt the neural Turing machines, which use the external memory matrix to augment memory ability. Furthermore, NSKT relates knowledge concepts (KCs) to related knowledge concepts (RKCs) as a whole to enhance the model's ability of skill awareness and improve prediction accuracy and prediction breadth. Experiments in the real-world KT datasets demonstrate that the NTM-based knowledge concept skill-aware knowledge-tracing model (NSKT) outperforms existing state-of-the-art KT models in modelling the students' knowledge state and discovering latent relevance among knowledge concepts.

For future studies, we will focus on mining hidden associations among knowledge concepts and building students' personalized answering paths in intelligent tutoring systems. Furthermore, we will construct the holistic structure of knowledge concepts to enhance students' understanding of how the overall knowledge affects each other.

## Figures and Tables

**Algorithm 1:**
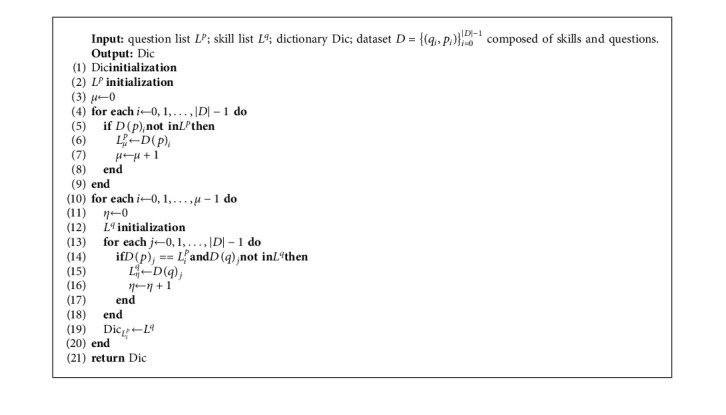
Question-skill dictionary algorithm.

**Table 1:** Related work.

Models	Methods	Categories
IRT	Logistic model	Statistical model
BKT	Bayesian model	

DKT	Long short-term memory network	Deep learning models
DHKT	Long short-term memory network	
DKVMN	Memory-augmented neural network	
SAKT	Self-attention	

**Table 2:** Notations.

Notations	Description
*p*	Problem/question
*q*	KC (skill/concept)
*a*	Answer correctness to the knowledge concept (KC) *q*
*c*	Answer correctness to related knowledge concept (RKC)
*M*	Number of unique KCs in the KT dataset
*P*	Probability
*S*	The RKCs
*H*	Interaction sequence of a student: {*h*_1_,…, *h*_|*H*|_}
*D*	Dataset
*E*	Encoding
KC	Knowledge concept
RKC	Related knowledge concept
Dic	Dictionary

**Table 3:** Example of a student answering question 33 in skill-level online tutoring systems. Question 33 is composed of skill *s*11 and *s*21. The student answers *s*11 incorrectly three times *t*_1_ − *t*_3_ in succession. Even if he answers *s*11 correctly at the timestamp *t*_4_, his mastery of *s*11 is very poor and his mastery of *s*11's-related skill *s*21 is not good too, so it is very likely to answer *s*21 incorrectly, in fact, he answers *s*21 incorrectly at the timestamp *t*_5_.

Timestamp	Skill	Correctness
*t*_1_	*s*11	0
*t*_2_	*s*11	0
*t*_3_	*s*11	0
*t*_4_	*s*11	1
*t*_5_	*s*21	0

**Table 4:** The relationship between *a*_*t*_ and *c*_*t*_ in skill-level online tutoring systems.

timestamp	*q*_*t*_	*a*_*t*_	*S*_*t*_	*c*_*t*_
*t*_1_	*s*11	0	*s*21	0
*t*_2_	*s*11	0	*s*21	0
*t*_3_	*s*11	0	*s*21	0
*t*_4_	*s*11	1	*s*21	1
*t*_5_	*s*21	0	*s*11	0

**Table 5:** The statistics of the three datasets.

	Students	Skills	Questions	Interactions (K)	MIN^a^	MAX^b^	AVG^c^
ASSIST09	4,162	124	26,688	526^d^	0	3	0.316
ASSIST17	1,709	102	3,162	942	0	2	0.472
EdNet	784,309	189	13,169	962	0	6	1.388

The symbol ^a^ indicates the minimum value of |*S*|, where 0 means that the question is a single KC (skill) question. The symbol ^b^ indicates the maximum value of |*S*|. The symbol ^c^ indicates the average value of |*S*|. The symbol ^d^K stands for a thousand.

**Table 6:** Test AUC results for all datasets (%).

	BKT	DKT-LSTM	DKT-NTM	DKVMN	SAKT	DSKT	NSKT
ASSIST09	72.06	84.45	84.53	84.37	84.70	84.88	**85.38**
ASSIST17	65.25	80.04	80.51	80.55	81.25	81.27	**82.35**
EdNet	66.28	78.91	79.49	79.72	79.83	79.71	**80.81**

Bold values indicate the best performance.

**Table 7:** Skill maps of ASSIST09 and *U*_1_'s interaction accuracy.

Skill index	Skill name	Accuracy (%)
32	Ordering positive decimals	100
33	Ordering fractions	100
37	Addition whole numbers	100
55	Absolute value	100

**Table 8:** Skill maps of ASSIST17 and *U*_2_'s interaction accuracy.

Skill index	Skill name	Accuracy (%)
2	Point plotting	30
4	Reading graph	100
34	Equation solving	50
95	Divisibility	30

## Data Availability

The datasets used to support the findings of this study are included within the article and are available from the corresponding author on reasonable request too.
